# *Helicobacter pylori* Infection of Gastrointestinal Epithelial Cells *in vitro* Induces Mesenchymal Stem Cell Migration through an NF-κB-Dependent Pathway

**DOI:** 10.1371/journal.pone.0029007

**Published:** 2011-12-28

**Authors:** Jonathan Ferrand, Philippe Lehours, Annie Schmid-Alliana, Francis Mégraud, Christine Varon

**Affiliations:** 1 Department of Bacteriology, Université de Bordeaux, Bordeaux, France; 2 Institut National de la Santé et de la Recherche Médicale, U853, Bordeaux, France; 3 Department of Immune & Inflammatory regulations, Université Nice Sophia Antipolis, Nice, France; 4 Institut National de la Santé et de la Recherche Médicale, U576, Nice, France; Institute of Microbial Technology, India

## Abstract

The role of bone marrow-derived mesenchymal stem cells (MSC) in the physiology of the gastrointestinal tract epithelium is currently not well established. These cells can be recruited in response to inflammation due to epithelial damage, home, and participate in tissue repair. In addition, in the case of tissue repair failure, these cells could transform and be at the origin of carcinomas. However, the chemoattractant molecules responsible for MSC recruitment and migration in response to epithelial damage, and particularly to *Helicobacter pylori* infection, remain unknown although the role of some chemokines has been suggested. This work aimed to get insight into the mechanisms of mouse MSC migration during *in vitro* infection of mouse gastrointestinal epithelial cells by *H. pylori*. Using a cell culture insert system, we showed that infection of gastrointestinal epithelial cells by different *H. pylori* strains is able to stimulate the migration of MSC. This mechanism involves the secretion by infected epithelial cells of multiple cytokines, with a major role of TNFα, mainly via a Nuclear Factor-kappa B-dependent pathway. This study provides the first evidence of the role of *H. pylori* infection in MSC migration and paves the way to a better understanding of the role of bone marrow-derived stem cells in gastric pathophysiology and carcinogenesis.

## Introduction

Almost all tissues possess progenitor cells which can evolve into different specialized cells under physiologic conditions. In addition, bone marrow-derived cells (BMDC) may take part in tissue repair in the case of chronic damage, making them good candidates for regenerative medicine [1]. Concerning the gastrointestinal tract (GIT), engraftment of BMDC can be detected in graft versus host disease or gastric ulcer patients, revealing a close relationship with the course of tissue regeneration [2]–[4]. The mice GIT can be repopulated in relation to the level of damage after local irradiation, as well as in the case of gastritis induced by a chronic bacterial infection [5]–[9]. Mesenchymal stem cells (MSC) are multipotent cells, able to migrate across tissues to differentiate into a variety of cell types depending on the surrounding microenvironment [10], [11]. MSC have been implicated in wound repair of numerous tissues, and the mechanism of trafficking has become clearer [12].

Besides their role as supportive cells in tissue repair, MSC could be at the origin of transformed cells in undifferentiated or Ewing's sarcoma, Barrett's esophagus and gastric adenocarcinoma [6], [13]–[16]. Concerning gastric adenocarcinoma, chronic infection of C57BL/6 mice with *Helicobacter felis* reproduces the classic sequence of histopathological events observed in humans infected by *Helicobacter pylori*, such as chronic gastritis, atrophy, metaplasia, dysplasia, and finally adenocarcinoma after a 15 month infection [17]. By using lethally irradiated C57BL/6 mice transplanted with gender-mismatched bone marrow from transgenic mice expressing bacterial β-galactosidase, the study of Houghton *et al.* suggests that *H. felis* infection results in a failure of local gastric stem cells to repair the injured tissue [6]. This loss of local stem cell repair may allow BMDC, suspected to be MSC, to engraft within the stem cell niche and assume the stem cell function by leading to a repopulation of the stomach with BMDC-derived epithelial cells. Once engrafted, and because of a bacterial persistence, BMDC are exposed to an infectious and inflammatory environment which is likely to drive their transformation [6], [17]. Our own results using a similar mouse model confirmed the participation of BMDC in the development of gastric dysplasia in *H. felis* and *H. pylori*-infected old mice [7]. MSC recruitment and transformation could be viewed together as one of the major factors in gastric carcinogenesis during Helicobacter infection, at least in the mouse model. Therefore, the different mechanisms involved in this multistep process have to be explored in greater depth, particularly the first step of MSC recruitment induced by Helicobacter infection.

Half of the world's 6.5 billion people harbor *H. pylori* in their stomachs and the infection is responsible for gastric cancer development in approximately 1% of them [18]. *H. pylori* strongly adheres to gastric cells and causes diverse cell damage via a major cytotoxin, VacA, and the CagA protein. *H. pylori* strains that harbor the *cag* pathogenicity island (*cag*PAI) encoding a functional type 4 secretion system (T4SS), induce the strongest Nuclear Factor-kappa B (NF-κB)-dependent production of pro-inflammatory chemokines, such as CXCL8, by a delivery of peptidoglycan to cytosolic NOD1 in epithelial cells [19], [20]. In mouse models, the cytokines involved in this inflammatory response are unclear as a CXCL8 homolog does not exist in the mouse. To date, no data are available concerning *H. pylori* infection and its ability to influence mechanisms of MSC migration, although the role of SDF-1 (CXCL12) and SCF have been suggested in the *H. felis* model [6]. MSC migration has been studied in various models and a large range of growth factors or chemokines has been identified as sharing chemoattractant properties *in vitro* [21]–[28]. Interestingly, a majority of these compounds have been shown to be over-expressed during *H. pylori* infection of epithelial cells both *in vivo* and *in vitro*, or associated with gastric cancer susceptibility, suggesting their potential role in MSC recruitment [29]–[37].

In order to better understand the role of MSC in *H. pylori*-induced gastric diseases and carcinogenesis, it is necessary to explore the first steps of the epithelial cell response to *H. pylori* infection in terms of the cytokine secretion which leads to MSC migration. In the present study, cytokines expressed by epithelial cells during infection were evaluated by qRT-PCR and ELISA, and the ability of the identified cytokines to induce MSC migration individually *in vitro* was tested. Finally, the role of the NF-κB signalling pathway in cytokine secretion was evaluated. This work demonstrates for the first time that the interaction of *H. pylori* with epithelial cells may lead to the migration of MSC by secretion of a combination of multiple cytokines, some of which are NF-κB dependent.

## Results

### Purification and characterization of murine MSC

**Figure 1:**
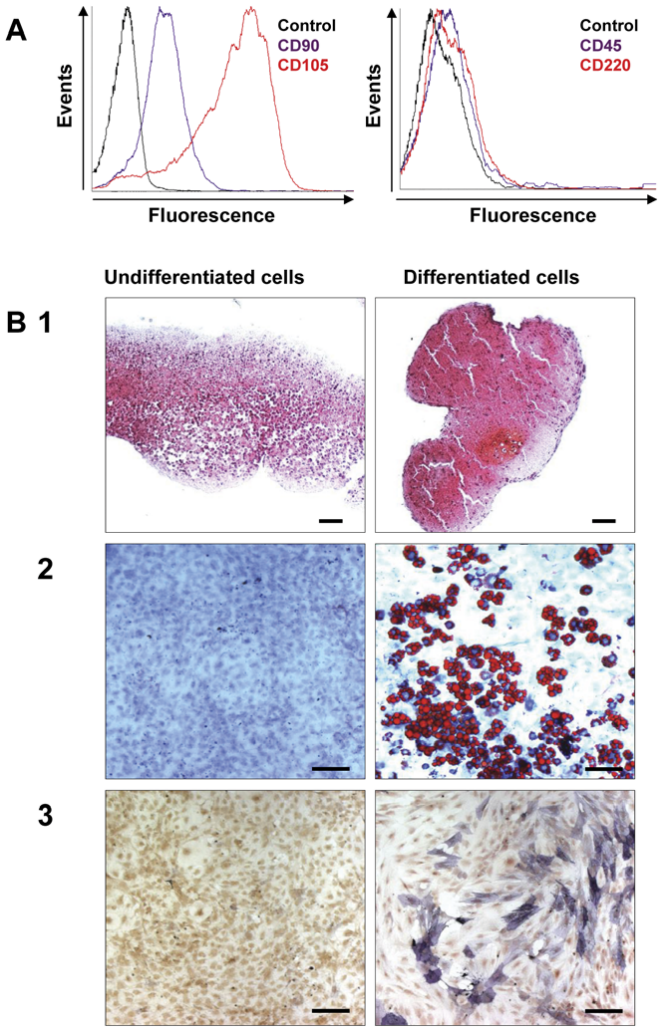
Characterization of murine MSC. A/ Immunophenotyping of murine MSC immunostained with antibodies against the indicated antigens (left panel: control as a black line, CD90 as a purple line and CD105 as a red line; and right panel: control as a black line, CD45 as a purple line and CD220 as a red line) after flow cytometry analysis. B1/ After 21 days of culture in chondrogenic medium, MSC were stained with Safranin O to reveal cartilage extracellular matrix. B2/ After 14 days of culture in adipogenic medium, MSC were stained with Oil Red O, a marker of lipid-rich vesicles. B3/ After 10 days of culture in osteogenic medium, MSC expressed elevated levels of phosphatase alkaline. One representative experiment out of three is shown. Original magnification ×20. Scale bar, 100 µm.

After *in vitro* amplification of adherent cells from C57/BL6 mouse femurs, CD90-sorted cells were characterized for MSC identity. The cells were positive for the mesenchymal markers CD90 and CD105 and negative for the hematopoietic markers CD45 and CD220 (Figure 1A). To validate MSC identity, a trilineage differentiation protocol was carried out [38]. After chondrogenic differentiation, cells stained by Safranin O expressed cartilage extracellular matrix (Figure 1B1). After adipogenic induction, cells displayed lipid-rich vesicles marked by Oil Red O staining (Figure 1B2). Finally, after osteogenic differentiation, cells expressed alkaline phosphatase (Figure 1B3). The ability of cells to differentiate into the three lineages defined and confirmed the MSC nature of the purified cells.

### Migration of MSC following *H. pylori* infection of gastrointestinal cells

**Figure 2:**
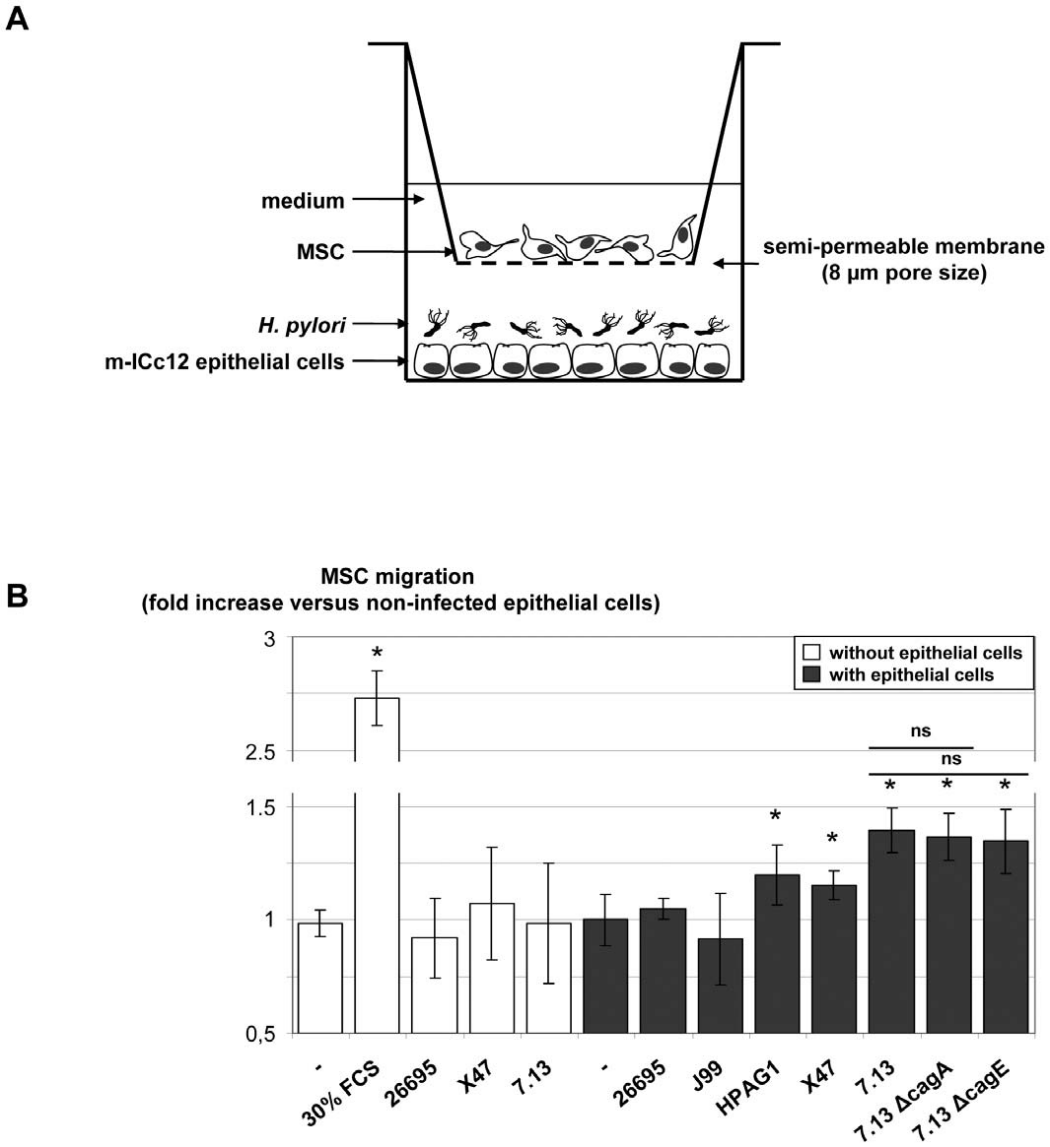
Migration of MSC following *Helicobacter pylori* infection of gastrointestinal epithelial cells. A/ After a 16 h coculture of epithelial cells with *H. pylori*, MSC were added to a cell culture insert with a semi-permeable membrane (8 µm pore size). MSC were allowed to migrate for 6 h before being fixed and stained and the number of migrated MSC on the lower side of the filter was determined. B/ MSC migration was evaluated after 6 h coculture with (grey) or without (white) epithelial cells and in the presence or not of 30% FCS and *H. pylori* 26695, J99, HPAG1, X47, 7.13 WT, 7.13 Δ*cag*A and 7.13 Δ*cag*E strains. Results correspond to the mean of three independent experiments, each performed in triplicate, ± SD. *: p≤0.01 compared to non-infected epithelial cells. ns: non-significant.

The ability of mouse m-ICc12 gastrointestinal epithelial cells to induce MSC migration after an *H. pylori* infection was evaluated using cell culture inserts, a well described model for MSC migration studies. Briefly, epithelial cells were cultured in migration medium and infected by *H. pylori* strains overnight. Cell culture inserts with 8-µm pore size membranes containing MSC were then added and MSC migration through the microporous membrane was measured after 6 h (Figure 2A). The basal migration of MSC in the presence of non-infected epithelial cells was the same as that in the presence of the medium alone, showing that epithelial cells alone were not able to stimulate MSC migration (Figure 2B). On the other hand, MSC migratory capacity was confirmed in response to a 30% fetal calf serum (FCS) stimulation used as the positive control. Stimulation of MSC with *H. pylori* 26695, X47 or 7.13 strains alone did not stimulate MSC migration. However, infection of epithelial cells with *H. pylori* HPAG1, X47 and 7.13 strains significantly stimulated MSC migration, indeed infection of epithelial cells with *H. pylori* 26695 and J99 strains had no effect on MSC migration. These results suggested a strain-dependent response. To assess the role of bacterial virulence factors, and particularly those encoded by the *cag*PAI (the CagA protein and the T4SS component CagE which is necessary for T4SS assembly), knock-out mutants of 7.13, 7.13Δ*cag*A and 7.13Δ*cag*E strains were tested. Both knock-out strains led to a similar stimulation of MSC migration as that induced by the wild type (WT) strain, excluding a role for CagA and the T4SS in this mechanism. As none of the bacterial strains was able to stimulate MSC migration when tested alone, the results suggest that MSC migration was induced in response to chemokines specifically secreted by epithelial cells infected by some strains of *H. pylori* independently of the T4SS.

### Identification of the chemoattractant factors

**Figure 3:**
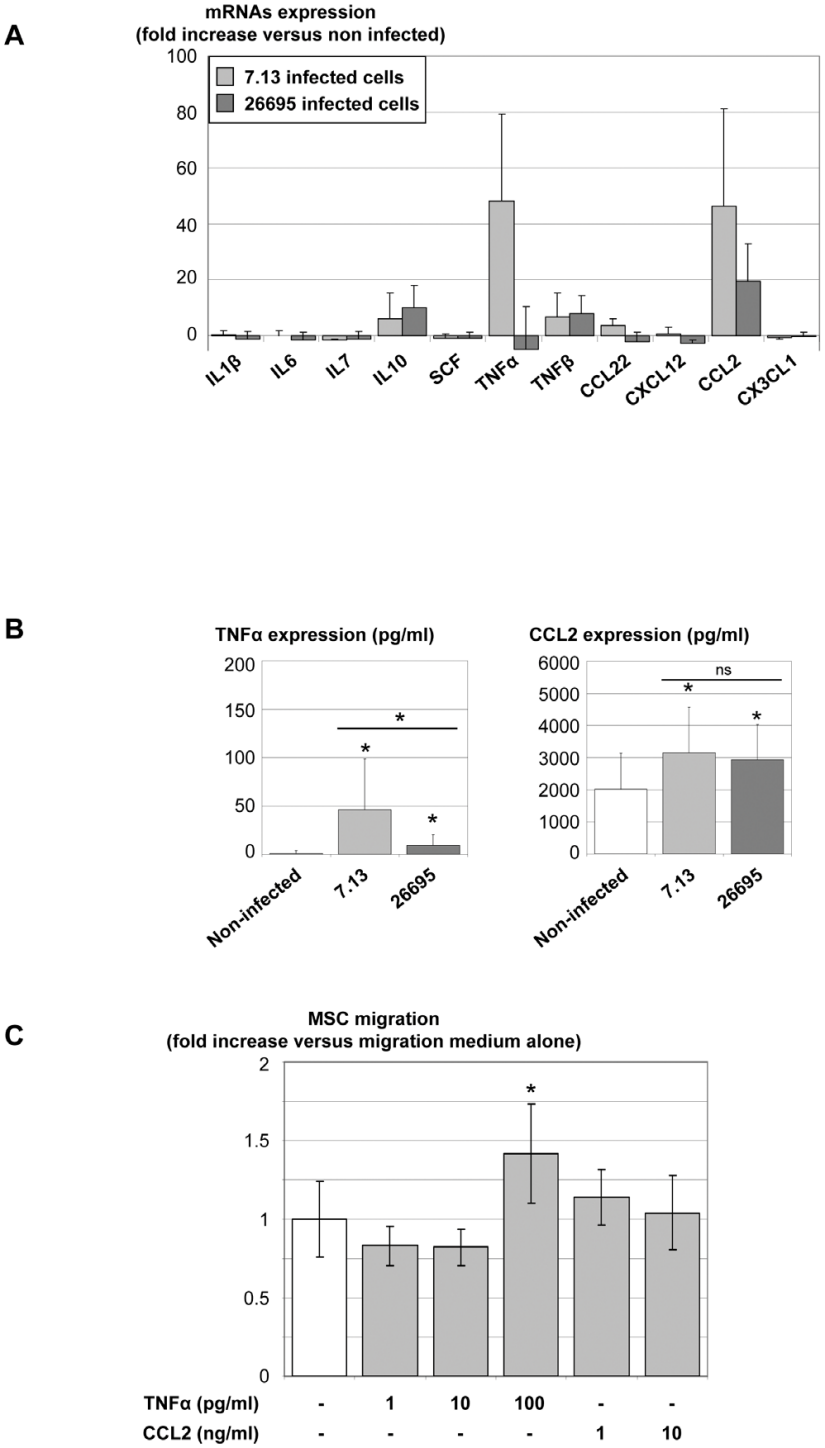
Epithelial cytokine expression and MSC migration. A/ After an 18 h coculture of epithelial cells with *H. pylori* 26695 or 7.13 strains in migration medium, mRNAs were extracted and mRNA expression of cytokines was measured by qRT-PCR. Results were expressed as fold increases compared to non-infected cells. Results correspond to the mean of 3 independent experiments ± SD. B/ Supernatants from non-infected (white bars), 7.13- (light grey bars) or 26695- (dark grey bars) infected cells were assessed for TNFα and CCL2 expression by ELISA. Results correspond to the mean of 4 independent experiments, each performed in triplicate, ± SD. * p≤0.05 compared to non-infected epithelial cells. C/ MSC were stimulated by increasing concentrations of purified cytokines and migration assays were then performed as described in figure 2. Results correspond to the mean of three independent experiments, each performed in triplicate, ± SD. *: p<0.01 compared to migration medium alone. ns: non-significant.

To identify the secreted molecules, epithelial cells were infected with either the “pro-migratory” *H. pylori* 7.13 strain or the “non-migratory” *H. pylori* 26695 strain as previously described. Cytokine mRNA expression of infected cells was measured by qRT-PCR 18 h post-infection and normalized with mRNA expression of non-infected cells. The cytokines tested were chosen as they are both over-expressed by infected epithelial cells and able to recruit MSC (IL-1β, IL-6, IL-7, IL-10, SCF, TNFα, TNFβ, CCL22, CXCL12, CCL2 and CX3CL1) (Figure 3A) [21]–[37]. Interestingly, the mRNA of two molecules, TNFα and CCL2, were largely over-expressed by m-ICc12 cells infected with *H. pylori* 7.13 with close to a a 50-fold increase compared to non-infected cells. This effect was also observed for CCL2 in response to the 26695 strain infection, but for TNFα it was observed at a very low level or not at all. The over-expression of TNFα and CCL2 by infected cells was confirmed in ELISA experiments (Figure 3B). The “pro-migratory” 7.13 strain induced a significantly higher increase in TNFα secretion compared to the “non-migratory” 26695 strain, suggesting a role for this cytokine in MSC migration induced by infection with the 7.13 strain. However, both 7.13 and 26695 strains induced a similar stimulation of CCL2 expression (Figure 3B). In order to clarify the role of TNFα and CCL2 in MSC migration, stimulation of MSC with various amounts of these cytokines in accordance with ELISA concentration results (TNFα: 1 to 100 pg/ml and CCL2: 1 to 10 ng/ml) was assessed in the migration model (Figure 3C). CCL2 alone was not able to stimulate MSC migration. However, 100 pg/ml of TNFα induced a significant increase in migration.

In reference to the work of Houghton *et al.* [6] proposing a role for CXCL12 in *H. felis* -induced MSC recruitment in mice, we also performed ELISA and migration assays with CXCL12, despite its low mRNA increase detected in qRT-PCR experiments. CXCL12 expression was not increased in response to infection with 7.13 or 26695 strains of *H. pylori* (Figure S1A). Stimulation with CXCL12 (1 to 750 pg/ml) did not stimulate MSC migration (Figure S1B). These results confirm that CXCL12 is definitely not involved in MSC migration in this model.

### Involvement of the NF-κB signalling pathway in MSC migration by *H. pylori* infected epithelial cells via TNFα secretion

**Figure 4:**
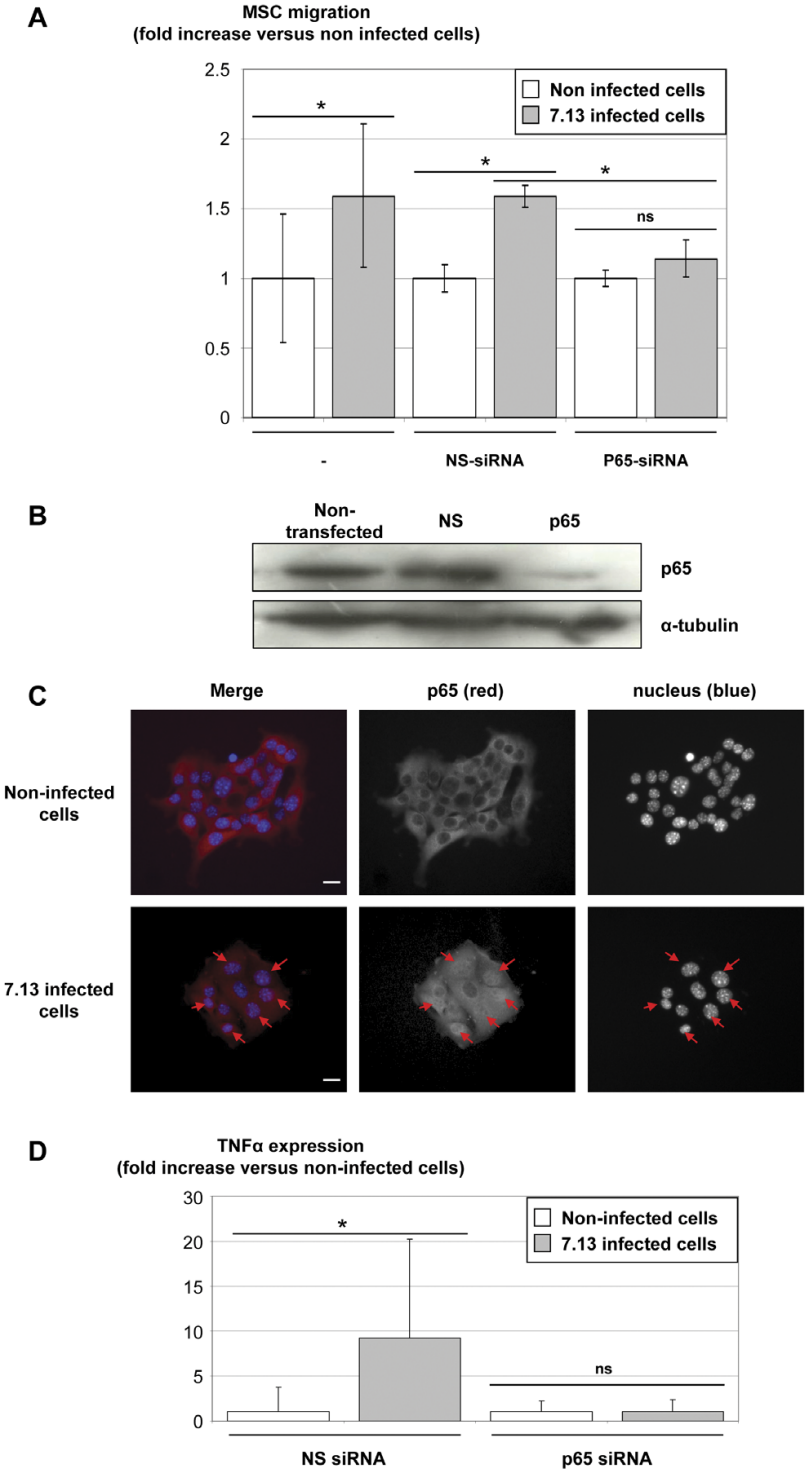
Infected epithelial cells induce MSC migration via a NF-κB-dependent TNFα secretion. A/ m-ICc12 cells were transfected with non-silencing (NS) or p65-siRNA prior to *H. pylori* infection. MSC migration assays were performed as described in figure 2 with non-infected epithelial cells (white) or 7.13 (grey) -infected epithelial cells. Results correspond to the mean fold of three independent experiments, each performed in triplicate, ± SD. *: p≤0.01 versus epithelial cells alone. B/ Transfection and silencing efficiency of p65- vs NS-siRNA were determined by western blot analysis for p65 expression 48 h post-transfection. Similar α-tubulin levels seen in all lanes are indicative of equal protein loading. One experiment representative of three is shown. C/ m-ICc12 cells were infected with *H. pylori* 7.13 strain and p65 translocation from the cytosol to the nucleus was assessed by immunofluorescent staining 4 h post-infection. The first vertical panel shows colored merge images with p65 immunostaining in red and nuclei staining (Hoechst 33342 compound) in blue, whereas black and white channels alone follow. Arrows indicate p65 translocation into nuclei (purple staining in the merge panel).White bar, 10 µm. One experiment representative of two. D/ Supernatants from NS or p65 transfected cells were assessed for TNFα after 7.13 infection (grey) or not (white) using ELISA experiments. Results correspond to the mean of two independent experiments, each performed in triplicate, ± SD. *: p<0.05 compared to the corresponding non-infected epithelial cells. ns: non-significant.

In order to investigate the role of the NF-κB signalling pathway, epithelial cells were transfected with control non-silencing (NS)-siRNA or NF-κB p65 subunit-siRNA and infected with *H. pylori* 7.13 strain. Transfection and silencing efficiency of p65- vs NS-siRNA were confirmed by western blot analysis for p65 expression (Figure 4B). Transfection with NS-siRNA did not modify the ability of the 7.13 strain to induce an MSC migration (Figure 4A). Conversely, p65-siRNA transfected cells lost the ability to induce MSC migration when infected with the 7.13 strain. The activation of the NF-κB pathway during a 7.13 infection was confirmed by immunofluorescent analysis showing p65 subunit translocation from the cytosol to the nucleus after a 4 h coculture (Figure 4C).

In order to determine the role of the NF-κB pathway in the production of cytokines responsible for MSC migration, TNFα expression was measured in the supernatants of NS- and p65-siRNA transfected epithelial cells. The TNFα over-expression during 7.13 infection which was observed in NS-siRNA transfected cells, was abolished in p65-siRNA transfected cells. These results confirmed the leading role of this cytokine in MSC migration and its NF-κB-dependent secretion by epithelial cells in response to *H. pylori* infection (Figure 4D).

## Discussion

MSC have been shown to take part in the gastric carcinogenesis process induced in response to Helicobacter infection, but the molecular mechanisms prompting their recruitment in gastric mucosa still remain unclear [6]. This study provides evidence that *H. pylori* infection of gastrointestinal epithelial cells is able to activate the migration properties of MSC, suggesting that a communication between *H. pylori* infected epithelial cells in the gastric mucosa and MSC exists via cytokine production. The two non-cancerous murine gastric epithelial cell lines currently available could not be used in coculture as they require permissive conditions at different temperatures to grow [39], [40]. Consequently, non-cancerous murine m-ICc12 intestinal epithelial cells were used as a suitable model of gastrointestinal epithelial cells [41]. m-ICc12 epithelial cells constitute a pertinent cellular model because of their gastrointestinal origin and because they do not correspond to a cancerous cell line but to normal immortalized cells, expressing chemokines in response to stimulation and particularly to *Escherichia coli* LPS [42]. This cell line is also responsive to Helicobacter infection and expresses NOD1 mRNA [43], [44]. In addition, we showed by immunofluorescence staining that *H. pylori* was able to adhere to m-ICc12 cells (data not shown).

Our results suggest that all *H. pylori* strains do not share the same capacity to induce MSC migration during infection of epithelial cells. Experiments using *H. pylori cag*A or *cag*E knock-out strains provided results indicating that this property is not correlated with the presence of the CagA protein or the presence of a functional T4SS, whereas the role of these bacterial pathogenic factors in carcinogenesis is well accepted. We hypothesize that the ability of *H. pylori* strains to induce *in vivo* migration of MSC is correlated to their ability to colonize and, consequently, to the appearance of cellular damage. Our *in vitro* results show that the most “pro-migratory” *H. pylori* strains induced the highest level of apoptosis of epithelial cells (Figure S2, File S1). These results suggest a potential link between the induction of cellular damage and the production of cytokines responsible for MSC migration. This relationship has to be compared to *in vivo* observations in which MSC recruitment was noted in damaged tissues during wound healing [5]–[9]. Finally, we hypothesize that epithelial cell apoptosis is one of the first steps necessary to activate MSC recruitment. Our study provides the first identification of cytokines which are secreted by infected epithelial cells and which play a role in MSC migration. TNFα and CCL2 have been shown to be over-expressed by epithelial cells infected with *H. pylori* strains. However, under our experimental conditions, only TNFα was able to induce an MSC migration when tested alone, with respect to the concentrations measured in supernatants of epithelial cells infected by “pro-migratory” *H. pylori* strains. Besides the leading role of TNFα, CCL2 may also play a minor role during MSC migration, as reported by others who claim that most cytokines are more effective on TNFα-primed cells [23]. Although these results may indicate the *primum movens* of MSC migration, this model did not include immune cells which, when recruited, may be strong producers of cytokines and also contribute to the MSC migration.

The NF-κB signalling pathway plays a major role in the ability of infected epithelial cells to induce MSC migration. Indeed, the NF-κB activation during *H. pylori* infection is required for TNFα secretion and MSC migration. In addition to the well known effect of the T4SS-injected peptidoglycan on NF-κB activation, numerous T4SS-independent molecules can stimulate the NF-κB pathway including those of pathogen-associated molecular patterns which activate toll-like receptor (TLR) signalling pathways, the TNFα-inducing protein (Tipα) or outer membrane vesicles [45], [46]. In humans, TLR2 and TLR5 are expressed by gastrointestinal cells and are activated by *H. pylori* infection which leads to NF-κB translocation and CXCL8 expression [47], [48]. The TLR specific *H. pylori* ligands have not yet been identified [49], as the classical ligands (i.e. LPS and flagella) seem to have limited effects [50]–[53].

The results obtained in this study allow a better understanding of the molecular mechanisms involved in the migration of MSC in response to *H. pylori* infection of gastrointestinal epithelial cells. Furthermore, the identification of a combination of cytokines including TNFα and CCL2, with TNFα as the most potent chemoattractant molecule, reinforces the theory initially proposed by Houghton *et al.* that Helicobacter-induced epithelial responses and damage can be directly responsible for MSC recruitment and homing in the gastric mucosa [6].

The role of MSC recruitment in the stomach still has to be clarified but mouse models suggest that complete units of dysplastic gastric epithelial glands are reconstituted with MSC-derived cells [6], [7]. We showed that MSC and gastrointestinal cells can fuse when cultured in close contact, conferring epithelial characteristics to MSC [54]. We hypothesize that *H. pylori*-infected epithelial cells are able to recruit MSC to balance *H. pylori*-induced apoptosis and assume stem cell function. Thereafter, MSC fusion with epithelial cells may render them more susceptible to transformation or could promote the cancerous process. Finally, we developed a model allowing the study of direct interactions between MSC and *H. pylori* infected epithelial cells in order to clarify MSC migration mechanisms. These results will now be helpful to study and understand cellular and molecular events, as well as bacterial pathogenic factors, involved in *H. pylori* and epithelial cell interactions leading to MSC recruitment and potentially implicated in gastric carcinogenic development.

## Methods

### Ethics Statement

Approval was obtained from the French Committee of Genetic Engineering (approval number 4608) and the local Central Animal Facility Committee of the University of Bordeaux before initiation of the study. All animal experiments were performed in level 2 animal facilities, in accordance with institutional guidelines as determined by the Central Animal Facility Committee of the University and in conformity with the French Ministry of Agriculture Guidelines for Animal Care.

Blood was obtained from the local Blood Bank “Etablissement Français du Sang Aquitaine Limousin” which collects blood anonymously and only blood that cannot be used for transfusion, i.e. use-by-date, was used to prepare blood agar for *H. pylori* culture. Informed consent is not applicable.

### Purification and differentiation of murine mesenchymal stem cells

To isolate adherent cells from bone marrow, femurs of male C57/BL6 mice were dissected and the ends of the bones were cut. The marrow was extruded by flushing the bone shafts with a 21-gauge needle filled with MesenCult medium (StemCell Technologies, Grenoble, France). All of the suspended cells from the two femurs were harvested and seeded in a 25 cm^2^ flask (BD Biosciences, Le Pont de Claix, France). After 48 h of culture, non-adherent cells were removed and the medium was changed three times a week. At confluence, cells were harvested with trypsin and diluted 1∶3 or 1∶4 at each passage. During culture, expression of CD45, CD90, CD105 and CD220 (BD Biosciences) was measured by flow cytometry (FacsCANTO, BD Biosciences). The cell population was sorted by flow cytometry according to CD90 expression (FacsARIA, BD Biosciences) in order to obtain a population positivity for CD90 of >98%.

Sorted cells were cultured in Dulbecco's Modified Eagle Medium (DMEM) supplemented with antibiotics (100 U/ml penicillin and 100 µg/ml streptomycin, all from Invitrogen, Cergy Pontoise, France) and 10% FCS, (Hyclone, Fisher Scientific, Illkirch, France).

For chondrogenic differentiation, 2.5×10^5^ cells were centrifuged at 600 g for 5 min. The resulting pellets were cultured in DMEM supplemented with 0.1 µM dexamethasone, 0.17 mM ascorbate-2-phosphate, 1% insulin-transferrin-sodium selenite supplement (all from Sigma, l'Isle d'Abeau, France) and 10 ng/ml of recombinant Transforming Growth Factor β3 (R&D systems, Lille, France). After 21 days of culture, the pellet was fixed overnight in 3.7% formaldehyde solution in phosphate-buffered saline (PBS), followed by paraffin embedding and cut into 7 µm thick serial sections. Slides were finally stained 5 min with 0.1% Safranin O (Sigma) in water and mounted with Eukitt (Labonord, Villeneuve d'Asq, France).

For adipogenic differentiation, cells were plated at a density of 8×10^3^ cells/cm^2^ and cultured for 3 days in DMEM containing 5% FCS (Invitrogen), 1 µM dexamethasone, 50 µM isobutyl-methylxanthine, 60 µM indomethacin and 100 ng/ml of insulin (all from Sigma). For the next 11 days, cells were cultured in DMEM containing 10% FCS and 100 ng/ml of insulin. On day 14, cells were fixed with 3% paraformaldehyde prepared in PBS for 10 min (Sigma), and lipids were stained with 1% Oil Red O (Sigma) in 70% isopropanol for 10 min and counterstained with thiazin solution (Diff-Quick II, Medion Diagnostics, Düdingen, Switzerland).

Osteogenesis was induced by culture at low density (2.5×10^3^ cells/cm^2^) in DMEM with 10% FCS, 10 mM β-glycerophosphate, 0.1 µM dexamethasone and 0.05 mM ascorbic acid (all from Sigma). On day 10, cells were stained after a 30 sec formalin-methanol fixation using the Leukocyte Alkaline Phosphatase Kit (Sigma) according to the manufacturer's recommendations.

### Epithelial cell culture

The murine m-ICc12 intestinal epithelial cell line was kindly provided by A. Vandewalle (INSERM U773, Paris, France) [41]. Cells were cultured in DMEM - nutrient mixture F12 (DMEM-F12) supplemented with 2% FCS, 20 mM HEPES, 20 mM D-glucose, 2 mM glutamine (all from Invitrogen), 5 µg/ml of insulin, 5 µg/ml of transferrin, 60 nM selenium, 50 nM dexamethasone, 1 nM triiodothyronine (all from Sigma), 10 ng/ml of EGF (Peprotech, Neuilly sur Seine, France), penicillin and streptomycin.

### Bacterial culture

*H. pylori* strains HPAG1 (kindly provided by L. Engstrand, Karolinska Institute, Stockholm, Sweden), as well as 26695, J99, X472AL (kindly provided by A. Labigne, Institut Pasteur, Paris, France) and knock-out strains of 7.13 (7.13 wild-type (WT), CagA deficient (Δ*cag*A) and CagE-deficient (Δ*cag*E), kindly provided by R. Peek, Vanderbilt University, Nashville, TN, USA) were used [55]. All *H. pylori* strains were cultured on Wilkins-Chalgren agar plates (Oxoid, Dardilly, France) supplemented with human blood (10% v/v) and antibiotics (10 µg/ml of vancomycin, 10 µg/ml of cefsulodin, 5 µg/ml of trimethoprim, and 10 µg/ml of amphothericin B) under microaerobic conditions.

For coculture experiments, *H. pylori* strains were grown at 37°C for 24 h, resuspended in PBS and adjusted to an OD_600 nm_ = 1 (corresponding to 2×10^8^ CFU/ml) in PBS before infection.

### Migration assay

Migration assays were adapted from a previously described protocol [23]. Briefly, migration was measured in cell culture inserts with 8-µm pore filters (BD Biosciences). The upper side of the filter was coated for 30 min at 37°C with 0.2% bovine gelatin (Sigma) in PBS. 100×10^3^ m-ICc12 cells were seeded on culture plates with 600 µl of medium. After overnight incubation, medium was changed and migration medium (DMEM supplemented with 1% FCS) was added. Cells were infected with the different strains of *H. pylori* at a multiplicity of infection (MOI) of 50. After overnight infection, 75×10^3^ MSC were added to the upper chamber of cell culture inserts. For assays with purified cytokines, 1 to 10,000 pg/ml of TNFα, CXCL12 (R&D systems, Lille, France) and CCL2 (Peprotech, Neuilly-Sur-Seine, France) in migration medium were added in the lower chamber. Migration observed in the presence of 30% FCS and with migration medium alone served as positive and negative controls, respectively. After 6 h of incubation and migration, filters were stained with Diff-Quick (Medion Diagnostics) according to the manufacturer's recommendations. Cells remaining on the upper face of the filters were removed with a cotton swab and washed with PBS. Filters were then cut out with a scalpel and mounted onto glass slides with Eukitt mounting medium. The number of cells that had migrated was counted using a ×20 objective and light microscopy. Each count was performed on 5 different randomly chosen fields per insert corresponding to a number of cells comprised between 50 cells (under basal conditions) and about 200 cells (under the positive control conditions). Each experiment was performed in duplicate.

### qRT-PCR

1×10^6^ m-ICc12 cells were seeded on 60 mm dishes and infected with 7.13 or 26695 *H. pylori* strains at a MOI of 50. Total RNAs were extracted using RNeasy kit (Qiagen, Courtaboeuf, France) and transcribed into cDNA using the Superscript III enzyme (Invitrogen). Real-time Polymerase Chain Reaction (RT PCR) was performed in an ABI PRISM 7900HT (Applied Biosystem, Courtaboeuf, France) and carried out using SYBR Green dye detection protocol. Amplification conditions were as follows: 50°C for 2 min, 95°C for 10 min followed by 40 cycles of 95°C for 15 s and 60°C for 1 min. Data were normalized for the amount of 4 housekeeping genes RNA (ACTB, GAPDH, HPRT and Ubiquitin) and mRNA expression was measured with the comparative C_T_ method (2^−ΔΔCT^). All gene-specific primers were in-house designed (Table 1).

**Table 1:** Primer sequences used for quantitative real-time polymerase chain reaction.

Target genes	Forward primer 5′-3′	Reverse primer 5′-3′
**IL-1β**	GAAGTTGACGGACCCCAAAAG	GTGCTGCTGCGAGATTTGAA
**IL-6**	ACAAGTCGGAGGCTTAATTACACAT	AAGTGCATCATCGTTGTTCATACA
**IL-7**	CATCATCTGAGTGCCACATTAAAGA	GGGCAATTACTATCAGTTCCTGTCA
**IL-10**	GGGTTGCCAAGCCTTATCG	CTTGATTTCTGGGCCATGC
**SCF**	CCCGAGAAAGATTCCAGAGTCA	CTGCTACTGCTGTCATTCCTAAGG
**TNFα**	AGGCGGTGCCTATGTCTCA	GGGTCTGGGCCATAGAACTG
**TNFβ**	ACTTTGTCTACTCCCAGGTGGTTTT	CGCACTGAGGAGAGGCACAT
**CCL22**	AGACCTCTGATGCAGGTCCCTAT	GCAGAGGGTGACGGATGTAGTC
**CXCL12**	GAGCCAACGTCAAGCATCTG	CGGGTCAATGCACACTTGT
**CCL2**	AGGCTGGAGAGCTACAAGAGGAT	ATCCAGGTTTTTAATGTATGTCTGGA
**CX3CL1**	CCGCGTTCTTCCATTTGTGTA	GCTGATAGCGGATGAGCAAAG

### ELISA

The concentrations of TNFα, CXCL12 and CCL2 were measured in cell supernatants by using ELISA kits in accordance with the manufacturer's protocols (TNFα and CXCL12 from R&D systems; CCL2 from Peprotech) on an ETIMax-3000 reader (DiaSorin, Saluggia, Italy). Supernatants were collected 24 h after infection, spun at 10,000 rpm for 10 min to discard bacteria and cell debris, and stored at −80°C until assayed.

### siRNA transfection

Two rounds of small interfering RNA (siRNA, Dharmacon, Lafayette, CO, USA) transfection into m-ICc12 cell lines was performed with double-stranded RNA (50 nM) as previously described [56], [57]. NS-siRNA (Dharmacon) with no known homology to mammalian genes was used as negative control.

For Western blotting, cells were collected in a reducing Laemmli sample buffer and lysates were sonicated and boiled at 100°C for 5 min. Equal quantities of protein (50 µg/lane) were subjected to sodium dodecyl sulfate–polyacrylamide gel electrophoresis (SDS–PAGE). Proteins were transferred from gels to nitrocellulose membranes (Amersham Pharmacia Biotech, Saclay, France) for immunoblotting with goat anti-p65 primary antibodies at 1∶1000 dilution (c-20, Santa Cruz Biotechnologies, Tebu-Bio, Le Perray en Yvelines, France). Proteins were detected by chemiluminescence (ECL+, Amersham Pharmacia Biotech) using horseradish peroxidase-coupled anti-goat secondary antibodies (DAKO, Trappes, France). The amounts of protein detected by western blotting were determined by scanning the autoradiograph, followed by data processing with NIH Image J software (1.37) (http://rsb.info.nih.gov/ij/).

### Fluorescent staining

25×10^3^ m-ICc12 cells were cultured on 12 mm glass coverslips for immunofluorescent staining. Cells were washed with PBS to remove cellular debris, then fixed with 3% paraformaldehyde prepared in cytoskeletal buffer for 10 min and processed as previously described [57]. Primary and secondary antibodies were diluted at the following concentrations: 1∶100 for goat anti-p65 antibodies, 1∶400 for Alexa-568-labelled rabbit anti-goat antibodies (Molecular Probes, Invitrogen); Alexa-488-labelled Phalloidin (1∶250) and Hoechst 33342 compound (1 µg/ml) were used as actin cytoskeleton and nuclear counterstains, respectively (both from Molecular Probes). Coverslips were washed in water and mounted on microscope slides with Fluoromount-G mounting medium (Clinisciences SA, Montrouge, France).

### Microscopy analysis

For immunofluorescent staining, cells were analyzed using an Eclipse 50*i* epi-fluorescence microscope (Nikon, Champigny sur Marne, France) equipped with Nis Element BR acquisition software and a ×40 (numerical aperture, 1.3) oil immersion objective.

### Statistical analysis

Quantification values represent the means of three or more independent experiments, each performed in duplicate or triplicate as indicated ± standard deviation (SD). Statistics were performed using the non-parametric Mann Withney test on SPSS16.0F software (SPSS Inc., Chicago, IL, USA).

## Supporting Information

Figure S1 **Study of CXCL12 expression in response to** ***H. pylori*** **infection and effect on MSC migration.** A/ Supernatants from non-infected (white bars), 7.13- (light grey bars) or 26695- (dark grey bars) infected epithelial cells were assessed for CXCL12 expression by ELISA. Results correspond to the mean of 4 independent experiments, each performed in triplicate, ± SD. * p≤0.05 compared to non-infected epithelial cells. B/ MSC were stimulated by increasing concentrations of CXCL12 and migration assays were then performed as described in figure 2. Results correspond to the mean of three independent experiments, each performed in triplicate, ± SD. *: p<0.01 compared to migration medium alone.
(TIF)

Figure S2 ***Helicobacter pylori*** **infection induced gastrointestinal epithelial cell apoptosis.** After a 48 h coculture of epithelial cells and *H. pylori* 26695, HPAG1 or 7.13 strains, cell DNA fragmentation was measured by the propidium iodide flow cytometric assay. Results correspond to the mean fold increase in three independent experiments performed in triplicate, *: p<0.01 compared to non-infected epithelial cells, #: p≤0.01 compared to 26695 infected epithelial cells.
(TIF)

File S1 **Apoptosis assays.**
(DOC)
