# Supplementary figures and images for: Helicobacter pylori Infection of Gastrointestinal Epithelial Cells in vitro Induces Mesenchymal Stem Cell Migration through an NF-κB-Dependent Pathway
Source: PLoS One. 2011 Dec 28;6(12):e29007. doi: 10.1371/journal.pone.0029007 (PMC3247220; doi:10.1371/journal.pone.0029007)

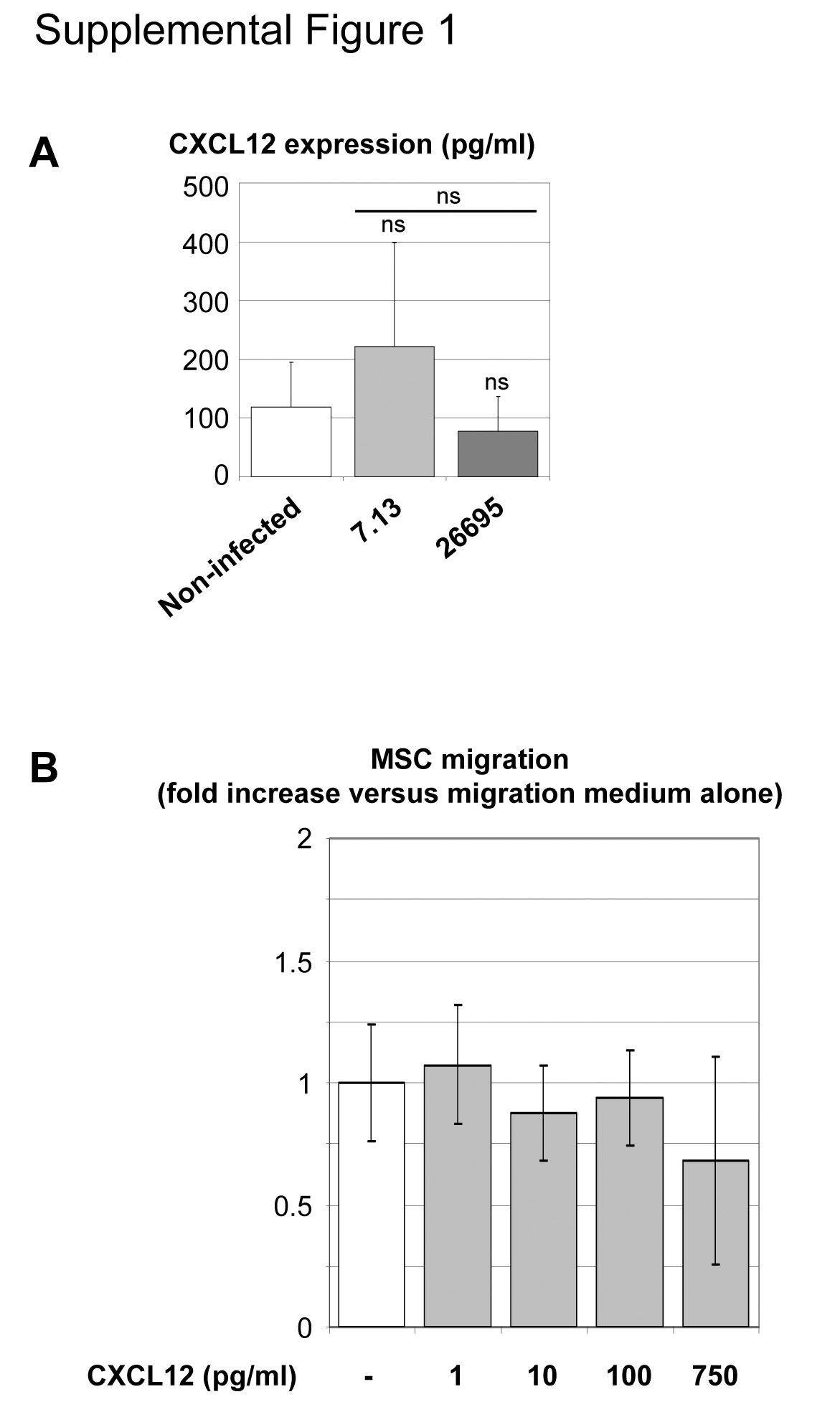

Supplement: Figure S1 — Study of CXCL12 expression in response to H. pylori infection and effect on MSC migration. A/ Supernatants from non-infected (white bars), 7.13- (light grey bars) or 26695- (dark grey bars) infected epithelial cells were assessed for CXCL12 expression by ELISA. Results correspond to the mean of 4 independent experiments, each performed in triplicate, ± SD. * p≤0.05 compared to non-infected epithelial cells. B/ MSC were stimulated by increasing concentrations of CXCL12 and migration assays were then performed as described in figure 2. Results correspond to the mean of three independent experiments, each performed in triplicate, ± SD. *: p<0.01 compared to migration medium alone. (TIF) [file pone.0029007.s001.tif]

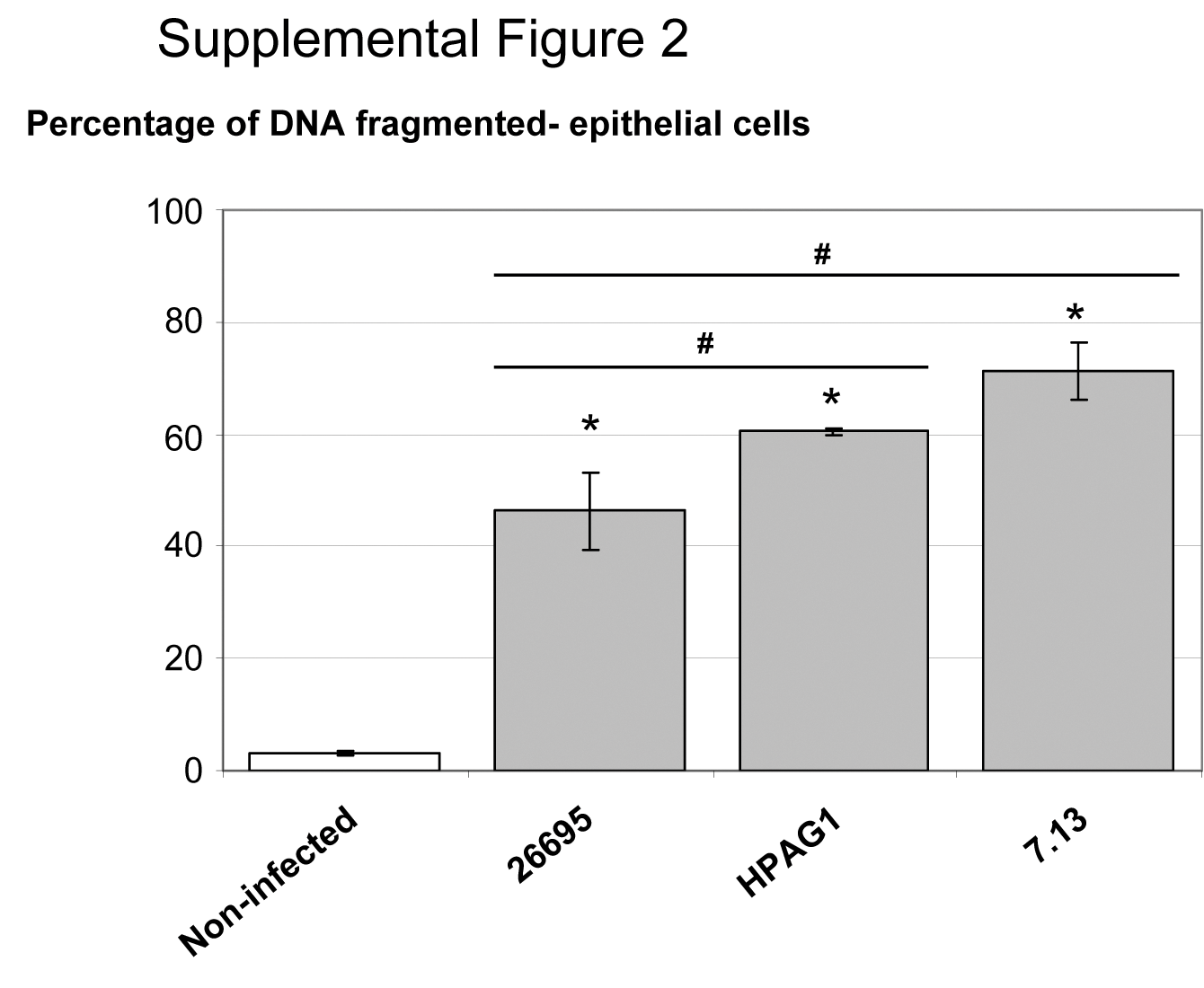

Supplement: Figure S2 — Helicobacter pylori infection induced gastrointestinal epithelial cell apoptosis. After a 48 h coculture of epithelial cells and H. pylori 26695, HPAG1 or 7.13 strains, cell DNA fragmentation was measured by the propidium iodide flow cytometric assay. Results correspond to the mean fold increase in three independent experiments performed in triplicate, *: p<0.01 compared to non-infected epithelial cells, #: p≤0.01 compared to 26695 infected epithelial cells. (TIF) [file pone.0029007.s002.tif]
