# Supplementary material for: Helicobacter pylori Infection of Gastrointestinal Epithelial Cells in vitro Induces Mesenchymal Stem Cell Migration through an NF-κB-Dependent Pathway
Source: PLoS One. 2011 Dec 28;6(12):e29007. doi: 10.1371/journal.pone.0029007 (PMC3247220; doi:10.1371/journal.pone.0029007)
Supplement: File S1 — Apoptosis assays. (DOC) [file pone.0029007.s003.doc]

**SUPPORTING INFORMATION S1**

**MATERIAL AND METHODS**

**Apoptosis assay**

Epithelial cell apoptosis during *H. pylori* infection was measured using a previously described protocol [1]. Briefly, 100x10^3^ m-ICc12 cells were seeded on 24-well plates in medium without antibiotics. After 24 h of culture, cells were infected with *H. pylori* at a MOI of 50. After 48 h of infection, infected cells and medium were harvested using trypsin and centrifuged at 2800 rpm for 5 min. Cells were resuspended in 100 µl of 0.1% sodium citrate, 0.1% Triton X-100 and 50 µg/ml of propidium iodide (all from Sigma). The samples were kept for 4 h at 4°C before reading by flow cytometry (FacsCANTO, BD Biosciences).

**REFERENCES**

1. Riccardi C, Nicoletti I (2006) Analysis of apoptosis by propidium iodide staining and flow cytometry. Nat Protoc 1: 1458-1461.
